# Regulation of the immune system by the insulin receptor in health and disease

**DOI:** 10.3389/fendo.2023.1128622

**Published:** 2023-03-13

**Authors:** Priya Makhijani, Paulo José Basso, Yi Tao Chan, Nan Chen, Jordan Baechle, Saad Khan, David Furman, Sue Tsai, Daniel A. Winer

**Affiliations:** ^1^ Department of Immunology, Faculty of Medicine, University of Toronto, Toronto, ON, Canada; ^2^ Buck Institute for Research in Aging, Novato, CA, United States; ^3^ Department of Medical Microbiology and Immunology, Faculty of Medicine and Dentistry, University of Alberta, Edmonton, AB, Canada; ^4^ Temerty Faculty of Medicine, University of Toronto, Toronto, ON, Canada; ^5^ Division of Cellular and Molecular Biology, Diabetes Research Group, Toronto General Hospital Research Institute (TGHRI), University Health Network, Toronto, ON, Canada; ^6^ Department of Laboratory Medicine and Pathobiology, University of Toronto, Toronto, ON, Canada; ^7^ Buck Artificial Intelligence Platform, Buck Institute for Research on Aging, Novato, CA, United States; ^8^ Stanford 1, 000 Immunomes Project, Stanford School of Medicine, Stanford University, Stanford, CA, United States; ^9^ Instituto de Investigaciones en Medicina Traslacional (IIMT), Universidad Austral, Consejo Nacional de Investigaciones Científicas y Técnicas (CONICET), Pilar, Argentina; ^10^ Leonard Davis School of Gerontology, University of Southern California, Los Angeles, CA, United States

**Keywords:** insulin resistance, immunometabolism, obesity, cancer, infection, inflammation, aging, pre-eclampsia

## Abstract

The signaling pathways downstream of the insulin receptor (InsR) are some of the most evolutionarily conserved pathways that regulate organism longevity and metabolism. InsR signaling is well characterized in metabolic tissues, such as liver, muscle, and fat, actively orchestrating cellular processes, including growth, survival, and nutrient metabolism. However, cells of the immune system also express the InsR and downstream signaling machinery, and there is increasing appreciation for the involvement of InsR signaling in shaping the immune response. Here, we summarize current understanding of InsR signaling pathways in different immune cell subsets and their impact on cellular metabolism, differentiation, and effector versus regulatory function. We also discuss mechanistic links between altered InsR signaling and immune dysfunction in various disease settings and conditions, with a focus on age related conditions, such as type 2 diabetes, cancer and infection vulnerability.

## 1 The insulin receptor in the immune system

Insulin is a vital hormone that controls the availability of glucose, required for nearly all cell processes (1). The cellular response to insulin has been heavily studied in liver, skeletal muscle, and fat cells due to their role in governing systemic nutrient availability. However, immune cells also rely on insulin for their metabolic needs and function, with activated and effector immune phenotypes exhibiting increased energetic demands (2). Insulin binding receptors, including the insulin receptor (InsR) and insulin-like growth factor 1 receptor (IGF1R), are expressed on cells of both branches of the immune system. Innate immune cells (e.g., monocytes, macrophages, neutrophils, and dendritic cells), as well as adaptive immune cells (e.g., T cells and B cells) express InsR/IGF1R at various stages of activation (3–5). In this review, we will discuss how InsR signaling modulates downstream immune cell processes impacting the overall immune response. Because insulin-mediated effects on immune cells, including insulin resistance, can drive both pro- and anti-inflammatory effects, we will also discuss proposed mechanisms behind insulin’s pleiotropic effects and highlight their implications in disease.

### 1.1 Overview of the insulin signaling pathway

Insulin signal transduction pathways downstream of the phosphorylated InsR/IGF1R have been studied in some immune cells. Insulin binding to the InsR and to the IGF1R promotes the uptake of glucose, its storage in the form of glycogen and lipids as well as its biosynthetic use (1). Both receptors dimerize and auto-phosphorylate at tyrosine residues upon insulin binding with differential affinities to the insulin ligand. Signal transduction events downstream of insulin binding have been extensively summarized previously (6). In brief, InsR/IGF1R signaling begins with the recruitment of insulin receptor substrates (IRS; IRS-1 through 6), with different isoforms enriched in different cell types. An IRS scaffold is generated leading to the formation of Src-homology 2 (SH2) domains capable of activating two branches of signal transduction: the Phosphatidyl inositol-kinase (PI3K)/Protein kinase B (PKB), or Akt pathway, and the Mitogen activated protein kinase (MAPK) pathway. Activation of P13K leads to phosphorylation of Akt at T308 *via* phosphoinositide-dependent protein kinase-1 (PDK1) and at S473 *via* mammalian (or mechanistic) target of rapamycin complex 2 (mTORC2). The complete phosphorylation of Akt drives activation of mTORC1 *via* the inactivation of Tuberous Sclerosis-1/2 (TSC1/2) (7). The activated mTORC1, in turn, activates ribosomal protein S6 kinase (S6K) and eukaryotic translation initiation factor 4E (eif4E) to exert the multiple cellular effects assigned to the complex.

Importantly, mTORC1 and Akt driven signaling cascades control downstream pattern-recognition receptor (PRR) expression, epigenetics, autophagy, and metabolic programs like glycolysis, pentose phosphate pathway (PPP), oxidative phosphorylation (OXPHOS), and glutaminolysis, in immune cells (8). Because mTORC1 and Akt also respond to nutrient availability, growth factors and stress signals, their potentiation by insulin signaling serves to couple energetic demands with immune cell function.

The response to insulin can take on distinct forms, for example, due to the context dependent use of IRS and Akt isoforms in each immune cell subtype (9, 10). Also, insulin can boost existing immune responses due to crosstalk with other pathways. For instance, in addition to mTORC1 mediated metabolic flux, InsR/IGF1R mediated Akt activation drives phosphorylation with numerous other target proteins, including inflammatory factors. For example, Akt activates the nuclear factor kappa B (NF-kB) pathway *via* degradation of inhibitor of kB (IkB) (11). In immune cells, NF-kB is a cardinal factor for activation, controlling maturation, proliferation and production of chemokines and cytokines such as CCL2 and TNFα. These molecules recruit and activate both innate and adaptive immune cells, orchestrating local and systemic inflammatory responses.

The second branch of InsR/IGF1R signal transduction triggered at the receptor/IRS complex is the MAPK-ERK pathway. The SH2 domains at the IRS scaffold also serve as docking sites for Shc adaptor proteins. Shc proteins activate the Ras-GTPase which then turns on the MAPK-ERK pathway. Activated ERK1/2 also has been recognized as a major pathway responsible for important consequences on immunity driving immune cell proliferation, metabolic re-programming, differentiation, inflammasome activation and cytokine production (12–15). Importantly, both MAPK and PI3K pathways promote glucose uptake *via* several glucose transporters (GLUTs), including GLUT3, affecting overall energy metabolism in immune cells (16). The cascade of insulin signal transduction *via* the PI3K and ERK pathways serves to create points of crosstalk and regulation of the insulin response. Insulin signaling thus potentiates other immune activation signals. However, it can also be dampened by several important negative feedback mechanisms. The activated S6K, IKKB and JNK proteins can block and degrade IRS-1 through serine phosphorylation of IRS-1/2 (17), modulating the InsR/IGF1R cascade at the earliest stage. Many of these proteins are also activated within inflammatory signaling pathways (e.g. TNFR-JNK) and contribute to insulin resistance (IR) (6). Together, this network of signaling events can both activate and attenuate inflammation in a context-dependent manner.

A third route of InsR signaling is through the recently described mechanism where InsR translocates into the nucleus and binds to promoter regions of key genes associated with immune cell recruitment (18). In this pathway, activated InsR colocalizes with host cell factor-1 (HCF-1) to mediate direct transcriptional activity (18). This phenomenon was primarily observed in cultured liver cells, and it remains to be seen whether a similar mechanism occurs in immune cells.

Insulin signaling is intricately tied to metabolic responses in immune cells as alluded to earlier. Thus, we will next discuss in-depth how insulin action interacts with these nutrient sensing pathways and metabolic responses to alter inflammatory states in innate and adaptive immune cells.

## 2 Insulin action in innate immunity

### 2.1 Macrophages and monocytes

Macrophages and monocytes are best recognized for their ability to sense the tissue environment and polarize to orchestrate an appropriate immune response at the site of infection or damage. Protein expression of both InsR and IGF1R has been reported on monocytes and macrophages (19, 20). *In vitro*, insulin treatment of mouse macrophages potentiated the LPS-mediated production of pro-inflammatory cytokines, IL-6 and TNFα, in a PI3K dependent manner (21), with a similar response in human monocytes (22). Several studies using myeloid lineage knockouts support that insulin signals are required for pro-inflammatory polarization. Indeed, myeloid lineage specific genetic ablation of InsR, IGF1R or IRS leads to the anti-inflammatory (or M2-like) polarization of macrophages (3, 23, 24), consistent with an overall pro-inflammatory (or M1-like) biasing by InsR/IGF1R signaling. Key studies on the direct effect of insulin on innate and adaptive cell types are summarized in **Table 1**.

**Table 1:** The acute effects of insulin and IGF1R signaling on immune cells by cell type.

Immune cell	Adjuvant effects of acute InsR/IGF1R signaling	References
Macrophages	Insulin drives increased production of inflammatory cytokines (IL-6, TNFα) in the presence of LPS/TLR activation InsR required for M1-like polarization	Tessaro et al. (21); Ratter et al. (22) Mauer et al. (3); Knuever et al. (23); Baumgartl et al. (24),
Dendritic cells	Insulin drives increased scavenger receptor expression via ERK signaling with and without TLR activation	Lu et al. (25)
Neutrophils	Insulin drives increased ROS formation via potentiation of the PI3K signal upon priming with N-formyl oligopeptide IGF1 delays apoptosis via PI3K signaling	Safronova et al., 2001 (26) Himpe et al. (27)
Eosinophils	Insulin drives increased peripheral eosinophil levels and mucus production in the lungs of healthy and diabetic mice upon ovalbumin allergen challenge.	Ferriera et al., 2017 (28)
ILCs	IGF1R in ILC3s supports differentiation and function against respiratory pathogens in neonatal lungs	Oherle et al. (29)
T cells	Increased IL-2 responsiveness and chemotaxis, improved glycolytic and mitochondrial metabolism, increased IFNy production is mediated by InsR Th17 polarization of CD4 T cells via IGF1R Insulin drives reduction of IL-10 production by regulatory T regs	DeBenedette and Snow (30); Berman and Center (31); Fischer et al. (32); Tsai et al., 2018 (5) DiTorro et al., 2020 (33) Han et al. (34)
B cells	Undetermined	–

**Figure 1:**
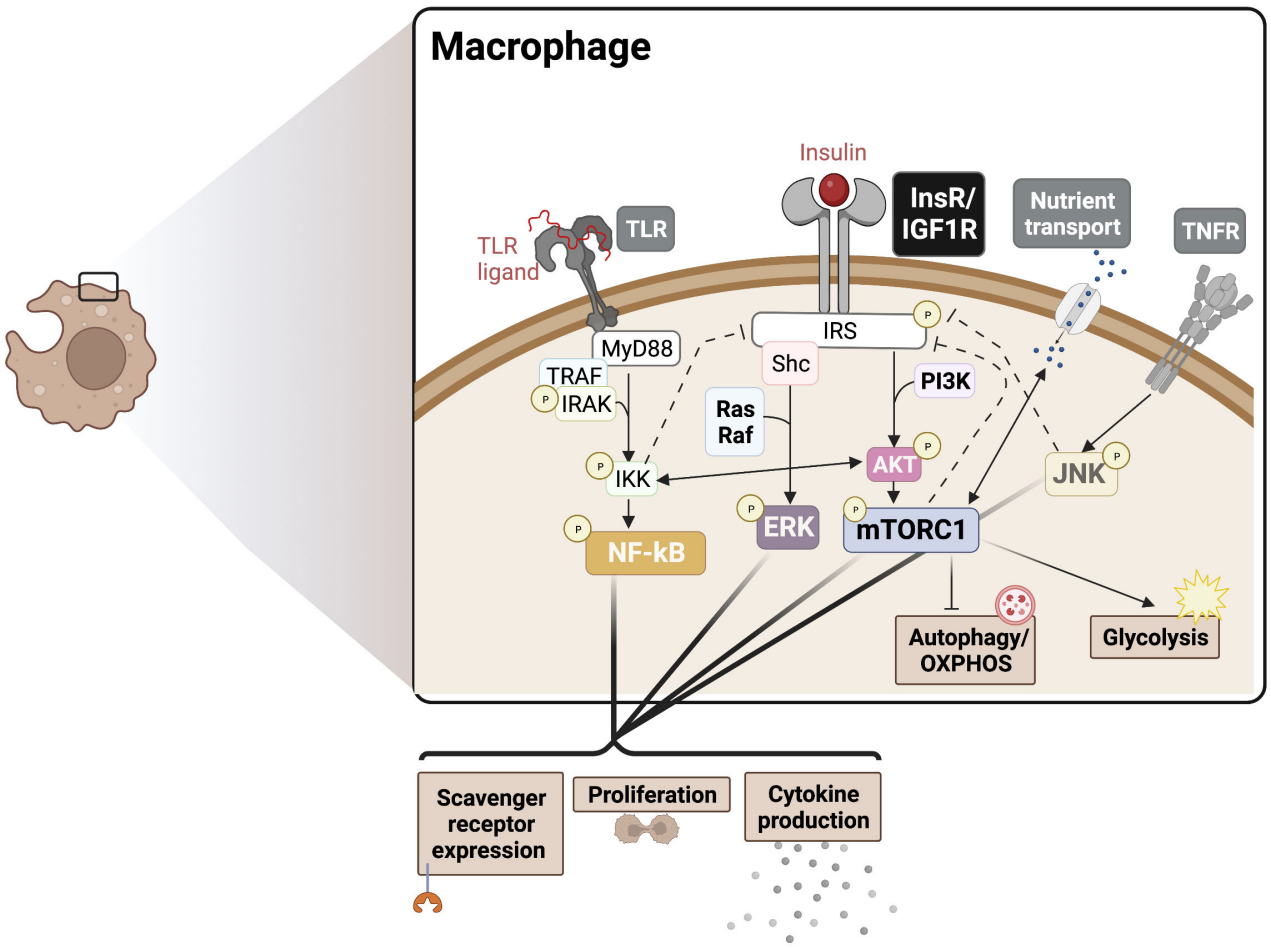
Insulin crosstalk with immune signalling pathways in macrophages. PAMPs/DAMPs trigger surface and endosomal TLRs, signalling largely *via* MyD88 to NF-kB to drive cytokine production, proliferation, and scavenger receptor expression. InsR/IGF1R signals through MAPK and Akt/mTORC1 to boost cytokine production and proliferation, supporting the TLR response. These signalling events also drive downstream metabolic programming to support the redox balance and energetic demands, promoting an inflammatory phenotype. Activation of mTORC1 also blocks autophagy, which provides substrates for OXPHOS. Various points of crosstalk exist between InsR, PRRs, cytokine receptor, and nutrient sensing responses can also drive insulin resistance *via* negative feedback mechanisms (dotted lines), for example in cases of chronic hyperinsulinemia. The degree to which each intersecting pathway is stimulated likely determines the nature of the pleiotropic phenotypes seen in IR macrophages. PAMPs, Pathogen-associated molecular patters; DAMPs, Damage-associated molecular patterns; OXPHOS, Oxidative phosphorylation; PRR, pattern recognition receptor; TLR, Toll-like receptor; MyD88, Myeloid differentiation primary response 88; NF-kB, Nuclear factor kappa B; InsR, Insulin receptor; IGF1R, Insulin-like growth factor 1 receptor; ERK, extracellular regulated kinase; mTORC1, mammalian target of rapamycin complex 1.

It has been recognized that pro-inflammatory innate immune cells rely on glycolysis and the PPP to meet biosynthetic demands, while immature and regulatory immune cells rely more heavily on OXPHOS and fatty acid oxidation (FAO) for obtaining energy and to inhibit metabolic drivers of inflammation (36, 37). Consistently, inflammatory M1-like macrophages show increased glycolysis, PPP, inducible nitric oxide synthase (iNOS) activity, and fatty acid synthesis, required to meet the biosynthetic demands of activation and corresponding with pro-inflammatory cytokine burst (38). Also, anti-inflammatory M2-like macrophages show increased glutamine metabolism, associated OXPHOS, arginase-1 activity, and FAO (39). A major mechanistic mediator of insulin driven pro-inflammatory responses is the mTOR complex, which supports glycolytic metabolism (40). Therefore, we speculate that insulin driven mTORC1 activation provides metabolic support for M1-like polarization. In myeloid cells, lineage-specific deletion of Raptor, a specific component of mTORC1, elevated the M2 macrophage signature, consistent with the effect of TSC1 deletion (mTOR activating), which results in sustained mTORC1 activation and a corresponding decrease in the M2 signature (41). Insulin-mTORC1 axis is also expected to promote pro-inflammatory outcomes *via* the inhibition of autophagy. Autophagy contributes to OXPHOS *via* lipid catabolism, which is inhibited upon mTORC1 activation (42). The above studies suggest that insulin signaling may have dominant effects on preferential inflammatory pathways *via* boosting glycolysis, PPP, and fatty acid synthesis (**Figure 1**).

However, M2-like macrophages also require glycolysis up to a threshold level to meet energetic demands (43). Therefore, insulin signaling may also participate in immune tolerance in certain situations. A new understanding of macrophage polarization states across both pro-inflammatory and suppressive disease conditions supports the adaptation of a spectrum polarization model (44), especially in cases of chronic unresolving inflammation. This model allows us to better appreciate heterogeneous macrophage responses to insulin depending on disease context.

### 2.2 Neutrophils

Neutrophils perform the important function of trapping and clearing pathogens as well as damaged tissue by producing reactive oxygen species (ROS) and releasing neutrophil extracellular traps (NETs). *In vitro* priming of neutrophils with insulin prior to activation with N-formyl oligopeptide leads to increased formation of ROS suggesting crosstalk with formyl peptide receptor mediated PI3K signaling (26). Furthermore, PI3K signaling was required for other neutrophil functions like cytokine production, chemotaxis, and degranulation (45), which maybe similarly potentiated by insulin treatment. Additionally, IGFR1-PI3K signaling was shown to delay apoptosis of the normally short-lived neutrophils (27). The mTORC1 pathway also promotes the release of NETs *via* HIF-1α (46). This suggests that insulin-mediated mTORC1 activation may also drive NET formation, influencing the clearance of infection. The context-dependent activation and metabolic reprogramming of neutrophils *via* InsR/IGF1R requires further study, especially given the key role neutrophils play in wound healing and infection.

A cell type closely related to neutrophils is the myeloid derived suppressor cell (MDSC). Mainly studied in the context of cancer and wound healing, MDSCs are similar to alternatively activated neutrophils and function to drive immune tolerance (47). While MDSCs rely on Akt-mediated STAT3 and HIF-1α signaling to produce regulatory effectors like ROS, IL-10 and Arg-1 (48, 49), they also require autophagy for MHC downregulation and rely on OXPHOS metabolism (50). The impact of insulin on MDSCs biology is a new consideration, with very little known about the expression of InsR/IGF1R on MDSCs or the basic *in vitro* effect insulin has on this cell type.

### 2.3 Eosinophils

Eosinophils have been well-recognized for their response to allergens and helminth infection with accumulating evidence for their role in tissue regeneration upon injury (51). Activated eosinophils upregulate IGF1R and therapeutic targeting of IGF1R can attenuate eosinophil mediated inflammation (52), suggesting a possible role for insulin/IGF signals in eosinophil function. Similarly to other immune cells, glycolysis is increased upon activation in eosinophils (53). Correspondingly, insulin treatment leads to increased peripheral eosinophil levels and mucus production in the lungs of healthy mice upon ovalbumin allergen challenge (28). Furthermore, eosinophils play a key role in immune cell crosstalk. For example, activated eosinophils have been shown to produce IL-4 and IL-13 to sustain the M2-like suppressive macrophage phenotype (54), thereby promoting wound healing and fibrosis. More work is needed to understand the impact of insulin in these processes, such as whether insulin exposed eosinophils can feedback onto inflammatory macrophages to regulate tissue homeostasis.

### 2.4 Dendritic cells

Dendritic cells (DCs) perform the key functions of antigen capture and presentation to lymphocytes required for adaptive immunity. *In vitro* insulin treatment drives maturation and increased scavenger receptor expression in human monocyte-derived DCs in an ERK-dependent manner (25). Additionally, TLR activation in DCs and subsequent Akt-phosphorylation directly boosts hexokinase 2 (HK2) function, an enzyme which catalyzes the first step in glucose metabolism, leading to increased glycolysis and PPP-derived NADPH to fuel lipid synthesis (55). Consistent with its adjuvant functions in other immune cells, insulin likely intersects with TLR signaling at boosting phosphorylated Akt signals upstream of mTORC1, potentially contributing to overlapping inflammatory mediator induction in DCs. In plasmacytoid DCs (pDCs), a subset of DCs shown to be important in anti-viral responses and autoimmune pathophysiology, type I IFN production upon TLR activation requires mTOR signaling (56). This interaction suggests that insulin-mediated mTORC1 activation may also augment IFN-I production in pDCs, aggravating autoimmunity or boosting anti-viral responses.

Although the direct role of the InsR-mTOR pathway on DCs has not been studied, the role of mTORC1 on DC activation and maturation has been highlighted in various studies (57). The cDC1 subset is described to cross-present antigen to CD8^+^ T cells, while cDC2s present antigen to CD4^+^ T cells. Studies using mice with conditional knockout of Raptor in the DC lineage showed that mTORC1 supports glycolysis while impairing CD8^+^ T cell priming by cDC1s, with no effects on CD4^+^ Th2 priming by cDC2s (58). This effect suggests a likely role for insulin-mediated mTORC1 activation in inflammatory metabolic reprograming in DCs, while inhibiting autophagy-dependent functions, such as antigen presentation, in DCs in a subtype-specific manner. Fully understanding the complex effect of insulin on antigen presentation in DCs will provide critical insights into their role in orchestrating immune dysregulation in diseases with disrupted insulin homeostasis.

### 2.5 Natural killer (NK) and other Innate-like lymphoid cells (ILCs)

ILCs are a family of lymphocytes with the capacity to rapidly release cytokines and perform killing functions in response to injury or infection but lacking adaptive rearranging antigen receptors like traditional lymphocytes but with the capacity to rapidly release cytokines and perform killing functions in response to injury or infection. Three widely accepted groups of ILCs have been documented: ILC1, ILC2 and ILC3, which are classified into further subtypes depending on the tissue (59). Whether and how ILCs respond to insulin/IGF1 as seen in other innate immune cell studies with a key role for mTORC1 and PI3K pathways is mostly unknown. Natural cytotoxicity receptors, found on all ILC subtypes, signal *via* the PI3K pathway (60) and could potentially cross-talk with InsR to aggravate ILC-mediated cytotoxicity and inflammation. Group 1 ILCs include NK cells and mediate crosstalk between the tissue and the immune response with the capacity to produce inflammatory cytokines. In NK cells, mTORC1 driven glycolytic metabolism was shown to be required for activation, cytotoxicity, and maturation (61). Interestingly, autophagy was required for catabolic processes and inhibition of apoptosis in ILC1s upon cell stress (62). Thus, we speculate, that insulin may boost acute ILC1/NK cell activation through mTORC1, while insulin-mediated inhibition of autophagy, may be detrimental for ILC1 survival in the face of stress.

In contrast to ILC1s, ILC2s are producers of type 2 cytokines like IL-4, IL-13, and IL-5 and can also drive tolerogenic activation of other immune cells. ILC2s were found to require glycolytic metabolism for cytokine production mediated by mTORC1 upon activation (63). Interestingly, the baseline level of OXPHOS required to the sustain naïve ILC2s, was found to be greater compared to NK cells (64). Speculating on the pro-inflammatory role of insulin in other immune cells, we expect differential effects of insulin on the suppressive ILC2s versus on pro-inflammatory ILC1/NK cells due to their differential thresholds of OXPHOS at baseline.

ILC3s are a distinct population of ILCs that produce IL-22 and IL-17 and play a role in mucosal homeostasis and Th17 responses. In ILC3s, mTORC1 was required for sustained HIF-1α and RORγt expression and production of cytokines (29). Further, conditional *Igf1r* ablation in ILC precursors driven by the RORγt promoter, lead to aberrant ILC3 differentiation and poor defense against respiratory pathogens in neonatal lungs, implying crosstalk with resident IGF1 producing fibroblasts and a role for ILC3s in early-onset asthma (30). The role for insulin/IGF1 on ILC3s in other mucosal tissues like the gut remains to be studied considering its critical role in gut healthy and barrier function in sustaining metabolic homeostasis.

## 3 Insulin action as an anti-inflammatory effector in the innate immune system

In contrast to the large body of evidence supporting the pro-inflammatory role of insulin in innate immune cells, some studies suggest that insulin may also dampen inflammation in certain ways. For example, the response to insulin in obese subjects or purified immune cells led to reduced TLR activation and transcription of TLRs (65, 66). Further, PI3K-Akt also inhibits the transcription factor, forkhead box O1 (FOXO1), a downstream target of Akt which is involved in cell proliferation, energy metabolism and oxidative stress (67). In macrophages, deficiency of FOXO1 has been shown to promote alternative polarizaion *via* the inhibition of glycolysis (68) and downregulation of target gene *tlr4* (65). The reduction in FOXO1 and TLR transcription may serve to dampen insulin mediated inflammation.

Insulin is also expected to stimulate the anti-inflammatory factor Nrf2 *via* the ERK pathway (69). Among many other functions, Nrf2 blocks production of inflammatory effectors such as ROS and IL-1β (70), which may support the anti-inflammatory polarization of macrophages and neutrophils. As alluded to earlier, insulin-mTORC1 axis also inhibits autophagy required for antigen presentation by macrophages and DCs and thereby, the activation of the adaptive immune response (57). Outside of these cell intrinsic effects, insulin also drives a systemic reduction in glucose levels. Low glucose levels result in fewer advanced glycation end products (AGEs) leading to dampened RAGE-mediated NF-kB activation and reduced inflammation (71). To better understand the action of insulin on innate immunity, we will need to delineate the short- and long-term as well as dose-dependent effects of insulin exposure, as the immune response to insulin may be subject to negative feedback mechanisms, which may have evolved to prevent tissue damage and unresolving inflammation.

## 4 Insulin action in adaptive immunity

### 4.1 T cells

The adaptive immune system is highly specialized and orchestrates tumor surveillance, anti-microbial responses, and generates immunological memory. Notably, cells of the adaptive immune system also express the InsR, which rises in expression upon activation (72–74). This increase in InsR expression likely reflects the T cells’ increasing energetic and biosynthetic demands. While a limited number of studies ascribed an anti-inflammatory role to insulin (73), emerging data also support an important pro-inflammatory role of InsR signaling in optimal T cell-mediated immunity (5, 33, 75). Addition of insulin to cultured T cells *in vitro* promoted IL-2 responsiveness (31), and chemotactic activity (32), which was accompanied by increased nutrient uptake and glycolytic reprogramming necessary for optimal T effector function (5). Thus, T cells lacking InsR expression exhibited an impaired glycolytic and mitochondrial metabolism, and reduced both IFNγ production and antigen-specific expansion upon influenza infection (5, 33). Consistent with the role of InsR in promoting inflammation and immunity, InsR signaling has been shown to dampen regulatory T cell (Treg) function in the setting of obesity and aging (35, 76). Interestingly, mTORC1 inhibition during the contraction phase promoted CD8^+^ T cell memory formation and function (77, 78). A recent study showed that InsR is dispensable for an effective memory CD8^+^ T cell response (79), suggesting that the immunostimulatory effects of insulin may selectively act during T cell activation and effector differentiation stages where glycolytic metabolism is critical, but not during memory differentiation which is predominantly supported by mitochondrial fatty acid metabolism.

T cells also express IGF1R, which signals through the Akt-mTORC1 axis (80) to regulate Th17/Treg differentiation both *in vitro* and *in vivo* (34). *In vitro*, stimulation with natural ligands of IGF1R favored the polarization of naïve CD4^+^ T cells towards Th17 phenotype and away from Foxp3^+^ Tregs. This change was accompanied by upregulated HK2 transcripts and glycolytic and mitochondrial metabolism within *in vitro*-differentiated Th17 cells. Furthermore, *igfr1* deletion in CD4^+^ T cells reduced IL-17A-expressing CD4^+^ T cell levels, increased central nervous systems-infiltrating Tregs, and protected against experimental autoimmune encephalomyelitis (34). Of note, these findings are in contrast to earlier reports, where IGF-1 was shown to expand Tregs and protect against autoimmune disease models of T1D and EAE (81). Here, Treg-specific genetic deletion of *igfr1* led to the loss of IGF-1’s protective effects. The discrepancies could arise from differences in the differentiation stage at which IGF-1 signals are perceived, during the activation and differentiation of naïve T cells (34, 82), or in pre-activated/differentiated T cell subsets (80, 83). The type and dosage of IGF-1R ligands, and signaling through IGF1R vs IGF1R/InsR heterodimers, could also contribute to the observed inconsistencies.

### 4.2 B cells

B cells are primary mediators of the adaptive and humoral immune response, achieving their functions *via* cytokine production, antibody secretion, and modulation of other immune cells (84). The exact role of InsR signaling in B cells has not been elucidated. However, the InsR has been shown to be expressed on B cells (74, 85) and is speculated to activate downstream PI3K/Akt and MAPK pathways much like in other immune cells.

**Figure 2:**
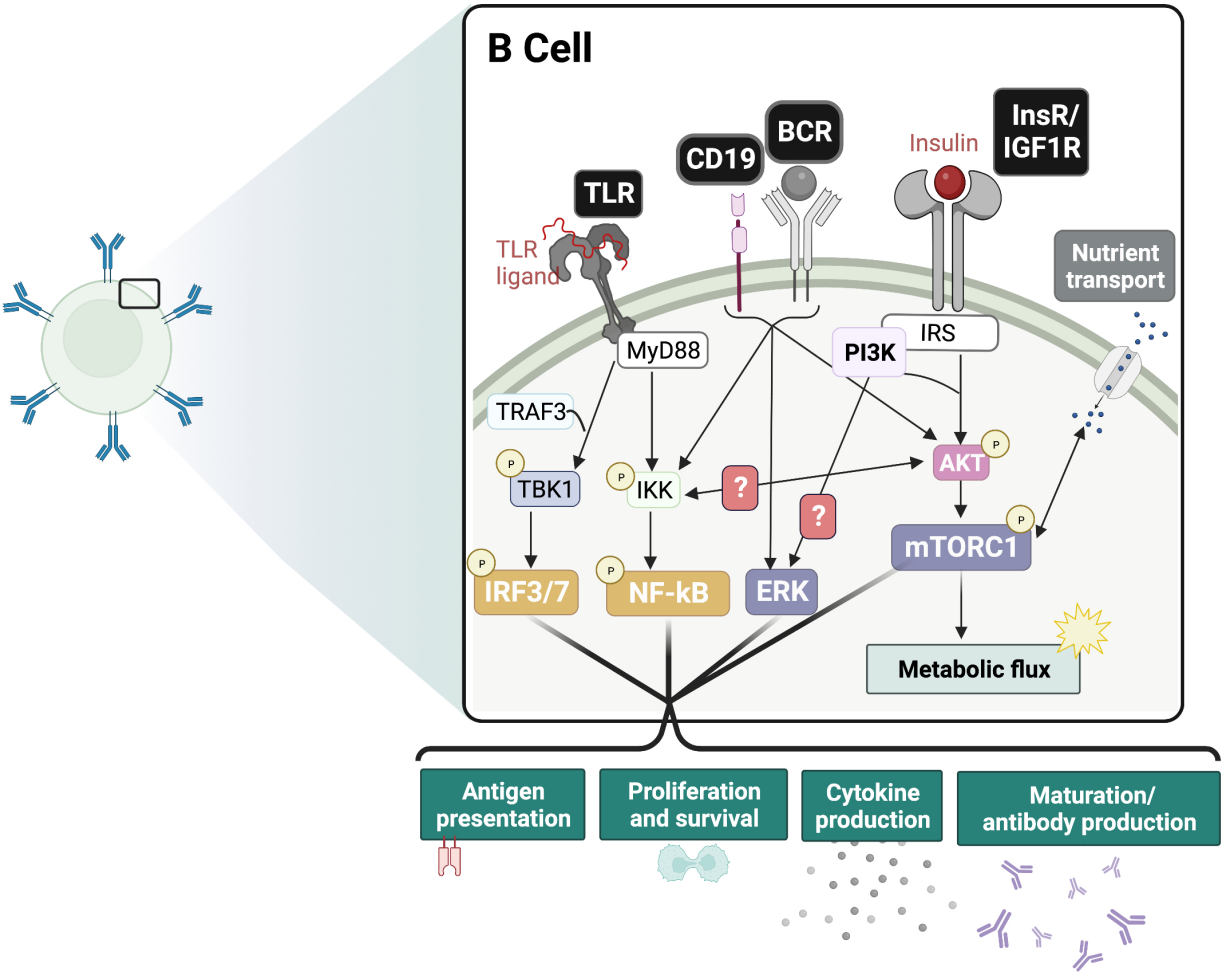
Insulin signaling functions as an adjuvant of B cell responses. Proposed crosstalk between InsR and BCR/CD19 and PRR signaling pathways. In the presence of antigen-BCR mediated ERK signaling and TLR-ligand mediated IRF and NF-kB signaling, the additive effect of InsR/IGF1 signaling is expected to drive inflammatory responses. This includes boosting glycolysis and nutrient transport, as well as maturation and antibody production, cytokine production, antigen presentation and proliferation. BCR, B cell receptor; TLR, Toll-like receptor; InsR, Insulin receptor; IGF1R, Insulin-like growth factor 1 receptor; mTORC1, mammalian target of rapamycin complex 1; ERK, extracellular regulated kinase; IRF, interferon regulatory factor; NF-kB, Nuclear factor kappa B.

During B cell development, PI3K signaling mediated by IL-7R and pre-BCR are required for the transition from the pro-B to pre-B and beyond the pre-B stages (86). In the peripheral B cell subsets, PI3K and Akt play a significant role in the specification of marginal zone (MZ) and B-1 cells (87–90). PI3K/Akt signaling is also crucial to B cell survival, activation, differentiation, and effector functions (**Figure 2**) (86, 91, 92). Cumulative PI3K signaling through tonic BCR signaling and BAFF-R is required for the growth and survival of peripheral B cells (93–96). Interestingly, the anti-diabetic drug metformin was found to attenuate BAFF-induced B cell proliferation and survival by inhibiting the mTORC1-PTEN/Akt-Erk1/2 signaling pathway (97). In the germinal center (GC) of peripheral lymphoid organs, PI3K plays a dual role. It is required for BCL6 expression, the transcription factor regulator of GC formation and differentiation of B cells into long-lived plasma cells (98), and maintenance of the GC reaction in B cells (86, 98–101). In contrast, PI3K also represses B cell identity, favoring plasma cell fate decision through IRF4 and Blimp-1 expression (101–104). These features are consistent with PI3K’s role in maintenance of the GC reaction especially in the light zone (105). Given this important role for PI3K in orchestrating GC reactions, it will be interesting to dissect how insulin and its receptor impacts such reactions in B cells.

Interestingly, PI3K signaling has also been shown to modulate class switch recombination (CSR), as pharmacological inhibition or B cell-specific genetic deletion of p110δ/α enhanced both IgG1 and IgE CSR in mice (106–108). Pten deletion, a negative regulator of PI3K signaling, also demonstrated significant reductions in IgG1 and IgE CSR (106). Together, IgG1 and IgE are mostly involved in type 2 immune responses, such as in the development of allergic Th2-mediated airway inflammation (109), and pharmacological blockade of p110δ PI3K has been suggested to be an effective treatment for allergies and atopic disease (108). More work is needed to determine if these pathways are similarly impacted by insulin signaling.

The role of Ras/MAPK signaling in B cells is well documented (110, 111). Erk signaling is necessary for the tonic BCR signaling required for immature B cells to differentiate into transitional and mature B cells (112). Erk was also shown to play a role in survival and maintenance of mature, antigen-specific IgG1^+^ B cells in the periphery (113). Erk is also necessary for the expression of Blimp-1 in plasma cells (114) and degradation of BCL6 (115).

In B cells, IGF1R expression is high in the pre-pro- and non-B cell bone marrow cells and decreases during differentiation, whereas InsR and IRS-2 expression increase during differentiation (74). The IGF1R was also expressed in the MZ and follicular (FO) splenic B cell populations. IGF1R deletion in B cells of chimeric mice showed no significant change in B cell development or peripheral subsets and did not affect BCR signaling. However, IGF1R deletion impaired T cell independent- but not T cell dependent-antibody response. The authors speculated that this effect was due to impaired IRS-PI3K signaling which regulates T cell independent responses (74). In patients with Grave’s disease, there was a subset of B cells with increased IGF1R expression which showed increased B cell proliferation and IgG production in response to IGF1 and CpG (TLR9 ligand) treatment (116). IGF1 has also been shown to increase IgG subtype production in human tonsillar and peripheral B cells in a CD40-dependent manner (117, 118). Interestingly, IGF2 was also shown to enhance regulatory B cells (Breg) proliferation and IL-10 production in response to specific antigen in an ovalbumin (OVA) allergic intestinal inflammation model (119), suggesting a role for IGFs in anti-inflammatory functions.

Although the effects of B cell InsR signaling have been less studied, there is likely significant metabolic overlap. Upon activation *via* BCR signaling, B cells rapidly increase glucose and amino acid uptake and glucose transporter GLUT1 expression in a PI3K-dependent manner (120–123). It is interesting to note that B cells rely less on aerobic glycolysis compared to T cells upon activation (120). Instead, BCR signaling diverts glucose catabolism away from glycolysis to: 1) the PPP to generate NADPH, crucial for neutralizing ROS and maintaining cellular redox status (121), 2) the *de novo* lipogenesis *via* ATP-citrate lyase to supply activated B cells with phospholipids for morphological changes (123), 3) the TCA cycle for efficient production of ATP through OXPHOS (120) and 4) the hexosamine pathway to allow for antibody glycosylation in long-lived plasma cells (124). Glycolysis appears to be dispensable for B cell activation and instead supports activation through redox maintenance through the PPP pathway, whereas OXPHOS and glutamine metabolism were shown to be indispensable for B cell growth and differentiation (125). Future studies using reductionist models will be important to tease out effects of insulin on B cell metabolism.

## 5 Insulin action on immunity in disease and aging

Given the recent advances summarized above, we propose that insulin plays a critical role in regulating both innate and adaptive immune cell function through fine tuning intrinsic metabolic programming. These findings have particularly important implications in diseases where insulin signaling is dysregulated, as such alterations could promote or potentiate the immune response. In the following sections, we discuss implications of altered InsR signaling in immune cells in disease states that are accompanied by inflammatory changes and how they contribute to shaping disease outcomes. Of note, while we focus on only a few conditions, it is plausible that any condition involving an immune response, including autoimmunity, would have relevant insulin-immune interactions.

### 5.1 Insulin on immune cells in obesity-related insulin resistance

A major driver of obesity-related IR is the establishment of low-grade chronic inflammation in metabolic tissues, including liver, muscle, and adipose tissue, especially visceral adipose tissue (VAT) (126). Within these tissues, immune cells such as macrophages and lymphocytes accumulate and produce pro-inflammatory cytokines, which in turn leads to IR and hyperinsulinemia. The role for hyperinsulinemia as a driving factor in this process is poorly understood, though previous work has implicated insulin as a global driver of VAT inflammation (127). Reduction of circulating insulin levels by approximately 50% in obese mice, using a diazoxide or streptozotocin (STZ) β-cell ablation regimen, led to a decrease in VAT macrophage expansion and a reduction in expression of pro-inflammatory cytokines/chemokines, such as CCL2, TNFα, IL-6, or IL-1β in VAT (127). Diet-induced obesity leads to accumulation of resident ILC1s by IL-12 production within the VAT which in turn drove pro-inflammatory polarization of macrophages *via* IFNγ production (128). However, eosinophils, which produce type 2 cytokines and promote M2-like states in macrophages, have been shown to have protective effects in obesity (54). Myeloid lineage-specific knockout of InsR protected against IR and associated VAT inflammation (3). In such studies with high-fat diet (HFD) fed mice, effects of reducing pro-inflammatory parameters in fat are seen in mice as early as 4 weeks of HFD. Thus it is likely that insulin is a critical driver of VAT inflammation early in disease (3, 24), potentially before marked changes in glucose levels occur.

#### 5.1.1 Insulin resistance in the immune system - an emerging paradigm?

Immune-mediated inflammation plays an important role in driving obesity-related IR (3, 21–24). We and others have shown that obesity is associated with increased infiltration of proinflammatory macrophages and adaptive immune cells in the liver, the intestines, and in the expanding VAT (84, 129–133), as well as a reduction in Tregs (35). On the other hand, this meta-inflammatory state is associated with an inability of the immune system to respond appropriately to certain foreign or tumor antigenic cues. In the context of anti-microbial immunity, functional defects in both humoral and cellular arms of the immune response have been observed across multiple species and disease models (134–137), although mechanistic insights are limited and inconclusive.

One possible mechanism that could explain these hallmark complications of diabetes might be that the cells of the immune system are also becoming insulin resistant themselves, leading to weakened overall effector function (138). This notion would raise the question of whether such cell-intrinsic IR underlies the observed immune impairments. Indeed, some work points to insulin being a new type of “metabolic adjuvant” to acutely boost immune cell action (5). For instance, mice in which the InsR is ablated specifically in T cells, a model of near complete immune cell IR, are vulnerable to viral infections such as H1N1 influenza due to poor T cell responses (5). Consistently, as we will discuss below, viral infections induce pro-inflammatory cytokine signaling which acutely reduces systemic insulin sensitivity in order to boost available insulin to immune cells for optimal anti-viral immunity (75). However, prolonged exposure to hyperinsulinemia would likely break this system if immune cells themselves became resistant to the action of insulin. This concept may have been especially relevant during the recent COVID-19 pandemic since obesity was a risk factor for poor outcome (139), as it was associated with weaker adaptive responses to clear the virus (140). While poor glycemic control associated with obesity also likely contributes to a dysfunctional immune response (141), studies from autopsied patients dying of severe COVID-19 show that insulin signaling pathways are amongst the most downregulated in tissue/immune cell infiltrated lung samples, linking local tissue insulin resistance to poor outcome (142, 143).

Some of the mechanisms of intrinsic immune system insulin resistance at the InsR are likely related to negative feedback mechanisms induced by hyperinsulinemia. Mechanistically, the accumulation of S6K and IKK, part of the mTORC1 and NF-kB pathways respectively, can degrade IRS-1 (11, 17). Because the IRS complex is required for early events in the insulin signaling pathway, negative feedback mechanisms targeting IRS may completely reduce the insulin response with overstimulation. In addition to built-in negative feedback mechanisms, disease-associated aberrant inflammatory and metabolic signaling can also promote the serine phosphorylation of IRS and dampen insulin sensitivity. For instance, inflammatory cytokines such as TNFα directly signal to impair insulin sensitivity (reviewed in 144). Elevated free fatty acids can also impair insulin-stimulated PI3K activation (145). These additional mediators of intrinsic immune cell insulin resistance may occur during both obesity and infection.

Another possible mechanistic consequence of intrinsic immune cell IR is differential use of Akt isoforms. Akt has three leading isoforms, of which Akt2 is the major isoform mediating insulin signals involved in glucose homeostasis (146). Greater levels of mTORC1 activity and lower Akt2 activation levels were observed in IR macrophages correlating with increased glycolysis (138). Accordingly, Akt2 deficiency increased M2-like signature, while Akt1 deficiency led to increased M1-like inflammatory signatures, supporting that Akt2 silencing can phenocopy insulin resistant macrophages in macrophages (138, 147). Differential effects of Akt1 and Akt2 have also been described in neutrophils and adaptive immune cells (148, 149).

Nonetheless, whether immune cells become insulin resistant with longstanding exposure to hyperinsulinemia and inflammatory stimuli is only beginning to be addressed. Exposure to insulin at supraphysiological levels and/or extended duration can lead to IR and altered immune cell function in culture studies. Culture with high levels of insulin inhibited Treg-derived IL-10, and impaired the ability of Tregs to exert cytokine mediated immune suppression, potentially explaining reduced Treg function in obesity (35). Culture of macrophages with high insulin levels led to macrophage-intrinsic IR and altered metabolic programming (150), which could impact functional polarization. In mice, both short term (7 days) and long term (10 weeks) HFD was sufficient to turn thioglycolate-elicited peritoneal macrophages (TEPMs) into an insulin resistant state. As mentioned, in macrophages intrinsic insulin resistance was linked to an unique state characterized by defective Akt2, coupled to sustained mTORC1 activation, increased glycolysis, but with a reduced M1-like cytokine profile and induction of M2-like regulatory genes (e.g. Arg1, Fizz1, Ym1) (138).

Less is known about whether adaptive immune cells can become insulin resistant with chronic obesity. As stated earlier, B cells are potential candidates since they express the InsR to significant amounts basally and upon stimulation. In contrast, it will be interesting to examine to what extent T cells, which only upregulate InsR upon activation, can become insulin resistant, and the metabolic and immune signals that promote IR in T cells during infection and obesity. Understanding how insulin signaling pathways intersect with BCR, TCR, and TLR signaling under longstanding obesity in terms of effector functioning is an important avenue of further work, as well as how it relates to immunological memory, including trained immunity (see below). Such studies may also give insights on observed complications of obesity or aging, such as heightened innate immunity to danger, coupled to reduced adaptive immune function.

On this topic, it has been shown that simply infusing insulin to better glycemic control may not necessarily benefit all patients with viral infection. In some cases, insulin infusions during COVID-19 was linked to cytokine storm in obesity (151, 152). This model would fit an immune cell IR hypothesis if sufficient insulin is infused to overcome immune cell-intrinsic IR, or if it elicits a form of trained immunity. If this renewed insulin signal occurs in innate immune cells, then an ensuing glycolytic burst associated with cytokine storm and mortality may occur. Interestingly, other glucose lowering therapies do not show this same effect (152), and they might potentially work also in part by relieving intrinsic immune cell IR themselves. These intricacies highlight the significant, potentially life or death implications, that may be learned from studying insulin action on the immune system. Thus, “immune system IR” is an important, but developing concept that still needs further study for its validation.

### 5.2 Cancer

Obesity fuels tumorigenic inflammation and alters cancer cell metabolism and growth (153). In addition, the growing tumor microenvironment presents challenges to the resident immune cells, such as hypoxia, nutrient deprivation, and the emergence of tumor-associated suppressive mechanisms. These challenges are further compounded by potential deleterious effects of obesity-associated hormonal and nutritional changes to dampen anti-tumor immunity.

In a mouse model of spontaneous pancreatic ductal adenocarcinoma (PDAC) with HFD-induced hyperinsulinemia, the reduction of insulin 2 gene expression (through *Ins2* heterozygocity) in the *Ins1* null background, led to reduced formation of early pancreatic lesions (154). The authors reported significant changes in immune cells in hyperinsulinemic PDAC-bearing mice using single cell sequencing analysis, with the greatest pathway alterations in B cells. Another reason for this protective effect, could be driven by insulin’s effect on the neutrophil-like, intratumoral MDSCs prevalent in early PDAC lesions (155). MDSCs require Akt-dependent phosphorylation of STAT3 for their suppressive activation (49), which is augmented by insulin signaling. In DCs, autophagy is required for antigen presentation (156). Autophagy inhibited by insulin-mTORC1 may also improve DC mediated cancer immune surveillance and antigen-specific responses explaining the protective effects in this model. While the immune cell intrinsic dysfunction caused by IR prior to PDAC remains to be fully understood, these studies suggest that insulin-mTORC1 driven inhibition of autophagy and Akt-STAT3 activation may drive tumor immune suppression *via* action on adaptive and innate immune cells.

These observations have important implications in metabolic syndrome, where loss of insulin receptiveness could differentially affect the ability of tumor infiltrating immune cell populations to compete with tumor cells for limiting nutrients and resources within the tumor microenvironment, leading to impaired anti-tumor function (157, 158). Additional investigative efforts are warranted to examine the role of insulin signaling and resistance in tumor-specific immunity.

### 5.3 Pre-eclampsia

Pre-eclampsia (PE) presents as high blood pressure and kidney damage and is correlated with the failure of immune homeostasis and increased inflammation at the maternal-fetal interface. Because both obesity and gestational diabetes are a pre-disposition to PE, researchers have studied the role of hyperglycemia and InsR/mTOR in the context of PE. Hyperglycemia resulted in the observed increase in AGEs in maternal blood and fetal tissues, which correlated significantly with an increased TNFα *via* the RAGE-NF-kB pathway (159). Moreover, as previously discussed, mTORC1 can dampen Treg responses which has also been suspected to invert the Th17/Treg balance in PE elevating responses to fetal antigens (160, 161).

### 5.4 Infection

In general, whole body IR increases the risk of infections, and infections also contribute to IR development and progression (75, 162). While the mechanisms underlying this association have recently begun to emerge, immunometabolism is particularly important in this context. Recent evidence suggests that T cells depend on insulin-induced signaling to respond against H1N1 influenza virus infection in mice by supporting T cell metabolic reprogramming and consequently, cell proliferation, activation, and cytokine production (5). Furthermore, since macrophages chronically exposed to insulin develop intrinsic IR and acquire a unique potentially anti-inflammatory phenotype coupled to an increased mTORC1-driven glycolytic profile (138), this undermines the resolution of infectious processes that requires pro-inflammatory macrophages. Neutrophils from diabetic rats also showed diminished phagocytosis and hydrogen peroxide production, which were recovered after insulin treatment (163). Although the impact of IR on metabolism in several immune cell subsets remains elusive, we anticipate that an impaired insulin sensitivity within immune and other cells alter cell phenotype and function, creating a favorable environmental condition for developing and progression of several infectious diseases.

The extent to which infection precedes and contributes to IR development is less clear. In general, it is plausible to consider that if the inflammatory status contributes to reduced insulin sensitivity, a sustained inflammatory response triggered by an infectious agent can also lead to IR (75, 164). In addition, specific therapies (e.g., steroids), hormones (e.g., cortisol), pro-inflammatory cytokines (e.g., TNFα, IL-1β, IL-6), nutrient status and type of pathogen may also potentiate or contribute to IR development. It is described that *Staphylococcus aureus* produces a protein (eLatS) with high affinity to insulin and blocks Insulin-InsR interaction, thus leading to IR (165).

The proposed bidirectional relationship between IR and infection has recently gained prominence with the severe acute respiratory syndrome-coronavirus-2 (SARS-CoV-2) (166). Various observational studies have found association between severe hyperglycemia and poor prognosis of COVID-19 (167). Noteworthy, COVID-19 patients without pre-existing metabolic-related diseases showed dysfunctional lipid and glucose metabolism and developed IR after discharge (168, 169). Moreover, another study described that both hyperglycemic and euglycemic patients hospitalized with COVID-19 showed impaired glucose metabolism and cytokine profile as well as developed IR at least 2 months after recovering from the disease (170). Whether reduced beta cell function as a result of SARS-CoV-2 infection is an additional contributor of these processes requires further examination (171). Induction of a transient insulin resistant, hyperinsulinemic state by a viral infection has been proposed as a physiological response to promote anti-viral CD8^+^ effector T cell activity (75). In the case of obesity, infection-induced persistent IR ultimately could be a maladaptive response if immune cells themselves also become IR, where deregulated glucose metabolism dampens antiviral immunity.

IR patients with uncontrolled blood glucose levels also have a greater risk of exacerbated COVID-19. Current studies showed that monocytes increase ROS-mediated HIF-1α stabilization which, in turn, enhance aerobic glycolysis during SARS-CoV-2 infection (172). These changes are associated with a glucose-enriched microenvironment, and they induce viral replication and cytokine storm, causing T cell dysfunction and lung epithelial cell death. The cytokine storm is also a hypothesized mechanism associated with COVID-19-related diabetes, since SARS-CoV-2 activates renin-angiotensin-aldosterone system (RAAS), which increases the production of IL-1α, IL-2, IL-6, TNFα, IFNγ, and MCP-1 and leads to pancreatic beta cell and systemic dysfunction (152, 173). Interestingly, insulin usage in patients in intensive care units is associated with increased COVID-19 mortality, possibly linked to increased glycolysis and associated cytokine storm, highlighting the importance and urgency of understanding pathological insulin action in the immune system (152). In the setting of chronic longstanding obesity and hyperglycemia, it is plausible that a high enough insulin dose could overcome intrinsic immune cell IR to facilitate increased glycolysis and cytokine storm, though more studies are needed to assess this possibility.

In general, blood glucose levels are reasonable predictors of disease severity in COVID-19 (174). Metformin, the most used antihyperglycemic drug, has also been prescribed as anti-inflammatory and immunomodulatory drug (175) with promising benefits to treat COVID-19. In addition to regulating glucose levels and increasing insulin sensitivity, metformin, as an activator of AMP-activated protein kinase (AMPK) and an endosomal pH modulator, could ameliorate disease through reducing the infectivity and survival of SARS-CoV-2 (176). Metformin also inhibits mitochondrial complex I and, consequently, oxidative stress and pro-inflammatory cytokine production, thereby promoting endothelial protection (173, 177). It will be interesting to determine if restoring insulin sensitivity inside IR immune cells during obesity is another mechanism of action by which metformin can boost immune cell clearance of SARS-CoV-2. Indeed, several studies have shown positive outcomes in metformin-receiving COVID-19 patients (178–180). However, the use of metformin must be avoided in patients with congestive heart failure and kidney diseases due to alterations in levels of other metabolic by products (181, 182).

More recently, the increased GP73 levels, a glucogenic hormone, were also correlated to SARS-CoV-2 infection in a mouse model, enhancing both glucogenesis and fasting blood glucose levels. The inhibition of GP73 was enough to restore the glucose levels, serving as another potential target to treat or avoid the progression to IR in COVID-19 (183). Overall, there is a bidirectional causal association between IR and infection. However, the mechanisms describing how infection leads to IR development, including the role of immune system metabolism in this context, still requires further investigation.

### 5.5 Insulin signaling and trained immunity

Emerging evidence suggests that the interplay between insulin and other pro-inflammatory signaling pathways contributes to trained immunity (reviewed in 184). Trained immunity is observed in innate immune cells, as a parallel to adaptive cell memory, and is defined as the long-term reprogramming by primary inflammatory insults that leads to an altered response to secondary challenges (185, 186). Innate cells are thought to achieve trained immunity *via* two interconnecting mechanisms: 1) biasing of metabolic circuits and 2) epigenetic memory established during primary exposure (185). Inflammatory stimuli like infections and vaccinations drive trained immunity *via* PRR driven activation of Akt-mTORC1 and NF-kB (185). The pro-inflammatory metabolic profiles these pathways drive remain active after the primary stimuli, despite complete resolution of inflammation, to drive more rapid pro-inflammatory responses to secondary stimuli (187). As previously detailed, InsR/IGF1R signaling also synergizes with Akt-mTORC1 and TLR-NF-kB activation. By boosting glycolytic cell metabolism and inflammatory cytokine production, insulin signaling also partakes in trained immunity.

In chronic obesity-associated IR, peripheral macrophages may take on an insulin resistant M2-like phenotype (138), while some adipose tissue macrophages, such as recruited types, display inflammatory M1-like polarization (188). The IR-dependent aberrant Akt signaling sustains mTORC1 activation resulting in enhanced glycolysis, which can occur acutely with insulin action or chronically during an insulin resistant immune state (138). Thus, both acute hyperinsulinemia driving Akt and mTORC1 activity, as well as an altered insulin resistant macrophage during chronic obesity with increased basal glycolysis and mTORC1 activity, might both facilitate downstream effects known to induce trained immunity (184). Some of these effects might be on mTOR-HIF-1α signaling, which alters metabolites to mediate histone modifications for trained immunity (189, 190), though more work is needed to dissect out pertinent metabolites in these settings. Further studies to test this idea are also needed to better delineate effects of insulin action and resistance in recruited vs resident macrophages, and in the setting of innate tolerance, which is considered distinct from trained immunity (186). This direction of work is especially important to reconcile observed phenotypes in immune function during chronic obesity, and whether these occur due to intrinsic immune system IR, such as discussed in the previous section on *Insulin Resistance in the Immune System - an emerging paradigm?* For instance, it may be possible that reduced insulin action cripples adaptive immunity but potentiates some forms of innate immunity through trained immunity.

The second key mediator of trained immunity is epigenetic memory, governing access to inflammatory gene loci. Systemic hyperglycemia was correlated with epigenetic marks like (e.g., H3K9me3 and H3K4me3) resulting in sustained NF-kB transcriptional activity (191). Elevated oxidized low density lipoprotein (OxLDL) levels observed in IR also promoted H3K4me3 methylation in monocytes driving NF-kB driven cytokine production (192). Epigenetic reprogramming at the hematopoietic stem cell level also leads to a higher myeloid to lymphoid ratio upon re-activation (193). These systemic consequences of whole body IR may also contribute to trained immunity (184).

### 5.6 Immune cell insulin signaling/resistance in aging

An emerging hallmark of aging is a state of chronic low-grade inflammation, which is also a characteristic of obesity. This state is known as inflammaging, and it is coupled to overall dysfunction, termed immunosenescence (194). Mechanisms fueling dysfunction of the aged immune system and onset of immunosenescence are of broad interest. As well it is of interest to identify biomarkers and develop therapeutic targets for age-related chronic disease.

Insulin signaling is an evolutionarily conserved axis that correlates negatively with longevity across several species, including *Caenorhabditis elegans* (195), *Drosophila melanogaster* (196), *Harpegnathos saltator* (197) and mice (198, 199). Notably, aging in mice is associated with a declined capacity for insulin clearance resulting in hyperinsulinemia (200). In humans, low plasma insulin levels and high insulin sensitivity have been attributed to improved survival and are key features observed in centenarians, suggesting a key role for insulin in regulating human lifespan (201).

Low level chronic activation of the InsR during aging might explain inflammatory shifts within the immune system that are observed with age, including the emergence of pro-inflammatory cell types (202). In the adaptive immune compartment, InsR engagement can constitutively inhibit FOXO1, a pro-longevity transcription factor whose deletion, along with FOXO3, in T cells heightens levels of pro-inflammatory cytokines, such as IFNγ or IL-17 (203). FOXO1 is also required to suppress a state of activation in CD8^+^ T cells, thereby allowing antigen-activated and expanded CD8^+^ T cells to maintain self-renewal and preventing their senescence (204–206). In *C. elegans*, genetic inhibition of DAF-2 (*C. elegans* InsR/IGF1R homolog) is shown to enhance immunocompetence, while DAF-16 (*C. elegans* FOXO homolog) is mainly responsible in delaying immune aging in DAF-2 mutants (195). These observations support the notion that a dysfunctional InsR/IGF1R pathway during aging might underlie the impaired immune responses of elderly individuals.

The interplay between aging and tissue infiltrating Tregs has also gained increasing attention over recent years. During aging, FoxP3^+^ Tregs secreting IL-10 are sustained by IL-6, a major cytokine of age, and accumulate across different tissues in old mice. This increase in Tregs may occur possibly to compensate for the low-grade unresolving inflammation associated with aging (207, 208). Single cell analyses have unveiled that aged Tregs possess an activated phenotype and display enhanced suppressive abilities compared to young counterparts, akin to those observed within tumors of patients with solid cancers (209). Interestingly, inside VAT, FoxP3^+^ Tregs accumulation has been shown to be associated with aging-related insulin resistance (210). Consistently, FoxP3^+^ Treg-specific InsR knockout mice aged to 52 weeks under normal chow diet were protected from the metabolic effects of aging. Improved metabolism in aged Treg InsR knockout mice were linked to significantly reduced numbers of both ST2^+^ and ST2^−^ Tregs in VAT, but not in brown adipose tissue (76). Interestingly, the VAT of the aged mutant mice also showed increased expression of inflammatory genes, such as *Ifng, Tnf, Il1b, and Tlr2*, implicating that some pro-inflammatory regulation can be favorable in restoring insulin sensitivity in age-associated glucose metabolism (76). Thus, insulin action on resident metabolic tissue immune cells is likely sufficient to dictate metabolic and tissue inflammatory outcomes with age.

Multiple drugs with anti-aging properties also link nutrient sensing to insulin action with potential ramification on immune function. Rapamycin, an anti-inflammatory drug inhibiting insulin-mTORC1 axis, has long been considered effective in extending lifespan (211), boosting immunity in the elderly (212), and delaying several age-related diseases (213–216). The mechanism of this immunostimulatory effect of rapamycin are not well characterized, but could be linked to inhibition of mTORC1-mediated S6 kinase negative feedback on age-associated chronic insulin signaling (5, 217). Similarly, metformin is another therapeutic that promotes anti-aging, pro-longevity, and improved health span outcomes by downregulating InsR-mTORC1 activity and the overall immunosenescence burden (218). The idea that rapamycin and metformin might improve insulin sensitivity within immune cells as one mechanism of immune rejuvenation remains to be validated.

Another set of anti-aging compounds, senolytic drugs, such as quercetin, dasatinib (Q+D) and fisetin, have shown protective effects against aging, and the ability to prevent adverse outcomes against infectious diseases, such as COVID-19 (219, 220). Interestingly, quercetin, may also have some capacity to block aspects of InsR action, though these effects were mostly seen *in vitro*, in higher doses. Nonetheless, this mechanism might be worthwhile to further investigate as an immunoregulatory agent to regulate nutrient-sensing networks during age-related metabolic disorders (221). Consistently, insulin infusion has been linked to cytokine storm, increased glycolysis and unopposed immune cell activation with COVID-19 (5, 152, 217). Senolytic drugs possess a “hit-and-run” activity, meaning that they reach maximal efficacy while minimizing side effects with only intermittent administration (222). This property might make senolytics a promising therapy over rapamycin for the aging population, considering that long-term rapamycin intake potentiates IR by inhibiting mTORC2 (223).

## 6 Conclusions and perspectives

Recent advances highlight the existence of an endocrine-immune axis, where insulin plays a critical role in regulating immune cell function and metabolism. However, much remains to be learned regarding the molecular basis of this endocrine-immune interaction. Given that multiple arms of the InsR signaling pathway converge with those downstream of antigen and costimulatory receptors, PRRs, cytokines and nutrient sensing pathways, future efforts are needed to tease apart the signaling networks insulin uses in specific immune cell types and how this is regulated in the face of different stimuli. We propose that Akt and mTORC1 are central regulatory hubs that integrate insulin signals with immune receptors and metabolic signals, given their established role in these pathways, both as a signal transducer/integrator and an off switch for negative feedback. This notion would fit with a model where homeostatic levels of insulin signaling allow for optimal immune responses, while perturbed signaling, such as during IR states where chronic inflammation and hyperinsulinemia foster immune cell-intrinsic IR, facilitates immune dysfunction. In the context of metabolic syndrome, systemic IR and associated hyperglycemia, dyslipidemia, and inflammation can act hand in hand with immune cell-intrinsic IR, coupled to chronic basal activation of danger signals like TLRs, to further dysregulate immune cell metabolism and functional outcomes. The net result would yield immune cells with a basal inflammatory tone, unable to boost its metabolism in the face of new antigen, which are hallmarks of immune dysfunction in obesity and aging. Whether aspects of trained immunity or tolerance (e.g. sustained TLR ligation) also exist during the insulin resistant state in innate immune cells during chronic obesity is an avenue of future research.

**Figure 3:**
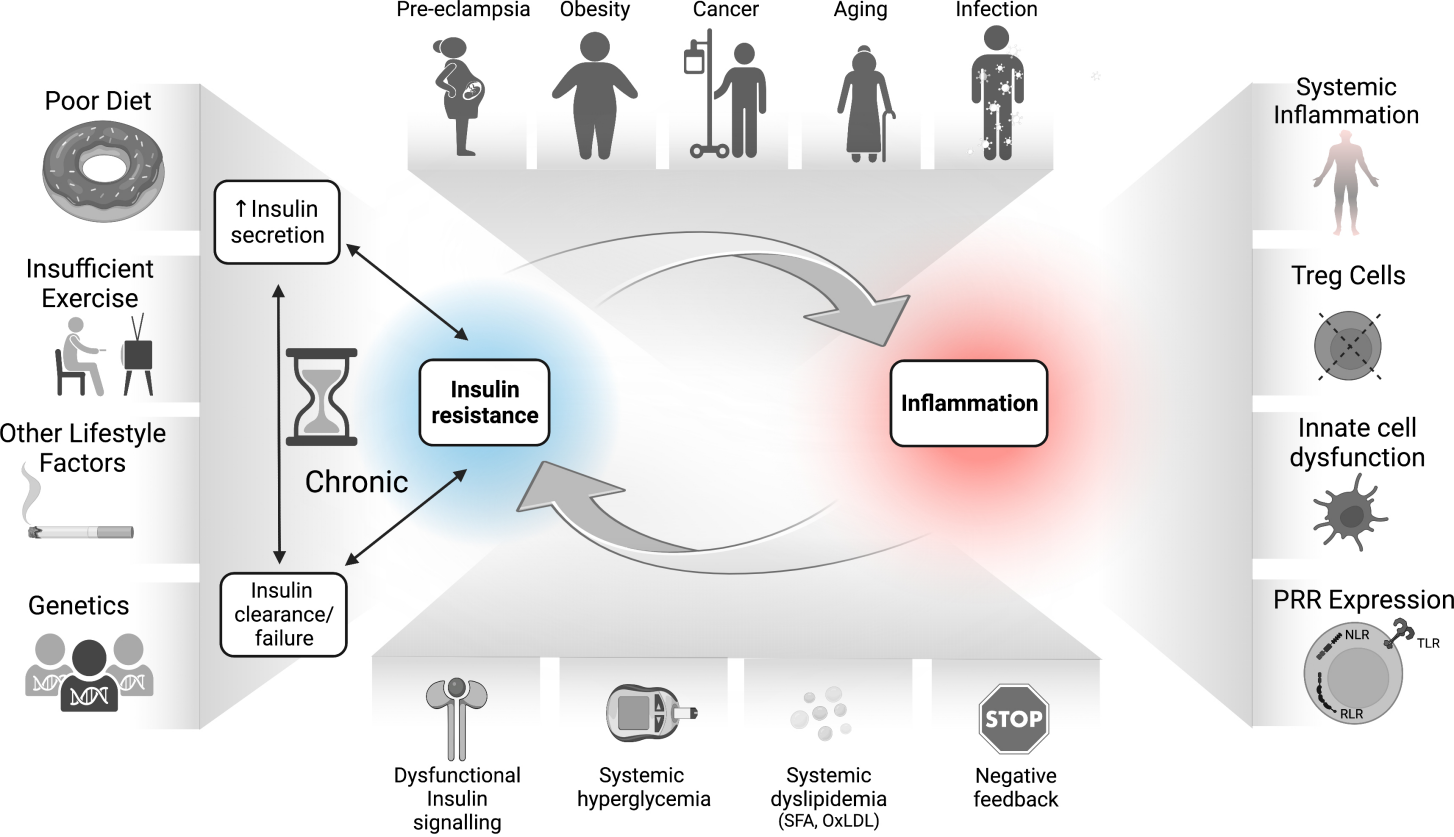
The bidirectional relationship between IR and inflammation in the pathogenesis of inflammatory diseases. Environmental, genetic, and dietary factors and their inflammatory responses can lead to hyperinsulinemia which drives dysfunctional responses in immune cells, including increased inflammatory tone and altered Treg function. The sustained IR-associated hyperglycemia and dyslipidemia also drive SFA and OxLDL production, increased RAGE activation, epigenetic changes, and accumulation of negative feedback signals on the InsR. Together with increased inflammation and PRR signaling, these immune/metabolic changes lead to whole-body as well as potentially immune cell-intrinsic IR. Immune cell IR in turn may undermine protective immunity and/or immune tolerance, which further exacerbates disease pathology. IR, insulin resistance; NF-kB, nuclear factor kappa B; mTORC1, mechanistic (or mammalian) target of rapamycin complex 1; NLR, nucleotide oligomerization domain (NOD)-like receptors; OxLDL, oxidized low density lipoprotein (LDL); PRR, pattern recognition receptor; RAGEs, receptor for advanced glycation end products; RLR, retinoic acid-inducible gene I-like receptors; SFA, saturated fatty acids; TLR, toll-like receptors; Treg, regulatory T cells.

As outlined in **Figure 3**, disrupted insulin-mediated metabolic homeostasis perpetuates a chronic meta-inflammatory loop that facilitates immune dysfunction. Conditions such as over-nutrition, aging, and pregnancy, as well as genetic and environmental factors could predispose individuals to developing IR, a state in which cells become impaired at perceiving insulin signals. A compensatory rise in insulin production to offset this impairment then leads to hyperinsulinemia and beta cell stress followed by functional decline. IR-associated nutrient imbalance, such as hyperglycemia and dyslipidemia, especially early in disease, together with potential intrinsic immune system IR, likely occurring later in disease progression, could thus drive dysregulation in immune cells with both pro-inflammatory and suppressive consequences, and contribute to the pleiotropic mechanisms of pathophysiology. These metabolic-immune interactions contribute to the pathogenesis of obesity, cancer, viral infection, pre-eclampsia, and age-related degeneration.

From a therapy perspective, the declined protective immunity in obese and/or aged insulin resistant individuals to infections and cancer raises the important question of whether and how insulin signaling pathway can be harnessed to rejuvenate the immune system. Strategies that dampen obesity or age-related inflammation would exert systemic beneficial, insulin-sensitizing effects. Insulin sensitizing agents such as AMPK agonists, including metformin, are potential candidates as they already are commonly prescribed medications in patients with T2DM, and mediate improved insulin sensitivity by increasing InsR tyrosine kinase activity, and increasing the recruitment and activity of the glucose transporters. In immune cells, this effect is expected to reactivate cell intrinsic insulin response.

Overall, immune cell insulin receptor signaling could represent a critical missing link in understanding manifestations and complications of obesity- and age-associated immune dysfunction. Considering the recurrent emergence of obesity, diabetes and aging as leading risk factors for poor clinical outcomes, especially in the context of severe respiratory infections, vaccination, cancer, and many other inflammatory diseases, a better understanding of the molecular basis of insulin-mediated immune regulation is imperative and may contribute to the design of new targeted strategies.

## Author contributions

DW, PM, PB, ST, YC, NC, and JB wrote parts of the manuscript, and/or generated figures/tables. SK and DF provided feedback. All authors had a chance to review the final manuscript. All authors contributed to the article and approved the submitted version.
